# Additive Effects of Clonidine Used in Propofol Sedation in Colonoscopy

**DOI:** 10.5812/aapm-156833

**Published:** 2025-02-25

**Authors:** Rabab Mohamed Mohamed, Ashraf Elsayed Elgahrib Abdalla, Mohsen M. Eissa, Reda Khalil Abdelrahman, Mohamed Galal Flefel, Asmaa Abdelbadie, Jehan Mohammad Ezzat Hamed Darwish

**Affiliations:** 1 Department of Anesthesiology, Surgical Intensive Care and Pain Medicine, Faculty of Medicine, Tanta University, Tanta, Egypt; 2 Department of Anesthesiology, Surgical Intensive Care and Pain Medicine, Faculty of Medicine, Al-Azhar University, Cairo, Egypt; 3 Department of Anesthesiology, Intensive Care and Pain Management, Faculty of Medicine, Al-Azhar University, Damietta, Egypt; 4 Department of Internal Medicine, Faculty of Medicine, Tanta University, Tanta, Egypt; 5 Department of Clinical Pharmacy, Faculty of Pharmacy, Nahda University, Beni-Suef, Egypt

**Keywords:** Colonoscopy, Sedation, Propofol, Clonidine, Patient Satisfaction

## Abstract

**Background:**

Propofol is commonly used for sedation during colonoscopy but often requires high doses.

**Objectives:**

This study aimed to compare the outcomes of propofol alone versus propofol combined with clonidine for colonoscopy sedation.

**Methods:**

In this randomized, double-blind controlled trial, 60 adult patients scheduled for elective colonoscopy were enrolled. Patients were divided into two groups: Group 1 (G1) received propofol alone, while group 2 (G2) received propofol plus 2 μg/kg clonidine intravenously over 10 minutes. Propofol infusion was initiated at 25 - 75 μg/kg/min IV for the first 10 - 15 minutes, then titrated to 25 - 50 μg/kg/min based on clinical response.

**Results:**

Sedation onset was significantly faster in G2 than in G1 (P = 0.001). The total propofol requirement was 22% lower in G2 (P = 0.001). Heart rate (HR) and mean arterial pressure (MAP) were significantly lower in G2 at induction and at the end of the procedure (P < 0.05). Patient satisfaction scores were higher in G2 (P = 0.042). The observer's assessment of alertness/sedation (OAA/S) score after induction was lower in G2 (P = 0.015), indicating deeper sedation. However, Aldrete scores in the post-anesthesia care unit (PACU) were lower in G2 (P = 0.001), suggesting a slower recovery.

**Conclusions:**

The addition of clonidine to propofol for colonoscopy sedation led to faster sedation onset, reduced propofol requirements, improved patient satisfaction, and deeper sedation, but with potentially prolonged recovery times.

## 1. Background

Colonoscopy is one of the most commonly performed procedures globally for the prevention, diagnosis, and treatment of various lower digestive tract conditions (1). Sedation is a crucial component that significantly enhances the procedure's efficiency and ensures patient comfort (2). The incorporation of sedation and analgesia in colonoscopy serves multiple purposes, including reducing patient anxiety and discomfort, enhancing procedure tolerability and patient satisfaction, minimizing complications, and establishing optimal conditions for assessment (3).

Propofol is a frequently used sedative for colonoscopy and can be administered alone or in combination with opioids or benzodiazepines (4, 5). However, using propofol as a sole agent often necessitates higher doses, potentially increasing the incidence of side effects. The decision to combine propofol with other analgesics or sedatives remains controversial, as the choice of drugs significantly influences procedural outcomes (6).

Clonidine, an α_2_ adrenoceptor agonist, has gained attention for its sedative and anesthetic-sparing effects. Its mechanism of action involves the stimulation of centrally located α_2_ adrenoceptors, with its analgesic properties primarily mediated through activation of these receptors in the dorsal horn of the spinal cord (7). The sedative effect of clonidine is dose-dependent and primarily targets the locus ceruleus, a small nucleus of neurons in the upper brainstem. Its sedative effect is not a consequence of hypotension or cardiovascular changes but rather a direct result of its action on the central nervous system (8).

## 2. Objectives

Given the potential benefits of combining sedative agents, this study evaluated the effectiveness, safety, and satisfaction outcomes of propofol alone versus propofol with clonidine in patients undergoing colonoscopy.

## 3. Methods

This randomized controlled trial was conducted on 60 adult patients aged 18 years or older, classified as American Society of Anesthesiologists (ASA) class I-II, who were scheduled for elective colonoscopy from June 2024 to September 2024 at Tanta University Hospitals, Egypt. Ethical approval was obtained from both institutional and regional committees (ID: 36264PR725/6/24) and registered with clinicaltrials.gov (ID: NCT06507410). Written informed consent was obtained from all participants. Exclusion criteria included individuals who had recently undergone a colonoscopy, had previous surgery to remove part of the colon, suffered from severe heart failure with an ejection fraction below 30%, had allergic reactions to propofol or clonidine, or required anesthetic drugs outside the study protocol.

### 3.1. Randomization and Blindness

Patients were allocated through computer-generated randomization with a 1:1 ratio (using https://www.randomizer.org/), stored in sealed envelopes to ensure unbiased group assignment. They were randomly divided equally into two groups of 30 each. In Group 1 (G1), participants received propofol alone. In group 2 (G2), participants received propofol plus clonidine. Patients and outcome assessors were blinded to the group assignments. A pharmacist not involved in the study prepared the interventional medications (saline 0.9% in G1 and clonidine in G2).

All patients underwent a preoperative assessment, which included a detailed history, a complete physical examination, and routine laboratory investigations. Baseline vital signs were recorded for 5 minutes before any intervention. Standard monitoring included electrocardiography, pulse oximetry, non-invasive blood pressure measurement every 3 minutes, and respiratory rate monitoring. Oxygen supplementation at a rate of 4 L/min was delivered using a nasal cannula. A single experienced colonoscopist conducted the colonoscopies.

In G1, sedation was induced with a continuous propofol infusion using a syringe pump. The initial rate was set at 25 - 75 μg/kg/min IV for the first 10 - 15 minutes, then gradually titrated to 25 - 50 μg/kg/min based on clinical response, with saline 0.9% administered intravenously over 10 minutes, 30 minutes prior to sedation induction. For G2, patients received the same regimen of propofol plus 2 μg/kg of clonidine intravenously over 10 minutes, 30 minutes prior to sedation induction.

Observer's Assessment of Alertness/Sedation (OAA/S) scores were recorded after the propofol induction dose by a trained observer. Hemodynamic parameters and blood oxygen saturation (SpO_2_) values were monitored 30 minutes before induction, at induction, and at the end of the procedure. Serious adverse events were defined and recorded, including significant changes in mean arterial pressure (MAP), bradycardia [heart rate (HR) < 50/min], apnea > 30 sec, and SpO_2_ < 85%.

After the procedure, patients were sent to a recovery area once their vital signs had stabilized. The recovery criteria included maintaining HR and MAP within 20% of the initial values, maintaining oxygen saturation above 90% while breathing room air, and being capable of standing without external support. The modified Aldrete scoring system was used at 15 minutes post-procedure for discharge, with a minimum score of 9 out of 10 required.

The five-item Likert scale was utilized for the evaluation of patient satisfaction (1, extremely dissatisfied; 2, unsatisfied; 3, neutral; 4, satisfied; 5, extremely satisfied) as well as symptoms of nausea and vomiting (9). Postoperative nausea and vomiting (PONV) were managed using a standardized protocol across both groups, which included ensuring adequate hydration (Ringer’s lactate at 4 mL/kg/hour) and administering ondansetron (4 mg IV) as a prophylactic antiemetic.

The primary outcome was the satisfaction scores. The secondary outcomes included comparisons of hemodynamic parameters and side effects such as nausea, vomiting, and psychological reactions during recovery.

### 3.2. Sample Size Calculation

The sample size calculation was performed using G*Power 3.1.9.2 (Universitat Kiel, Germany). An unpublished pilot study was conducted with five cases in each group, revealing that the mean (± SD) satisfaction score (the primary outcome) was 3.6 ± 0.89 in G1 and 4.4 ± 0.98 in G2. The sample size was determined based on the following considerations: An effect size of 0.898, a 95% confidence limit, 90% power of the study, a group ratio of 1:1, and an addition of 2 cases to each group to account for potential dropout. Consequently, 30 patients were recruited for each group.

### 3.3. Statistical Analysis

The statistical analysis was conducted using SPSS version 26 (IBM Inc., Chicago, IL, USA). Quantitative parametric data were expressed as mean and standard deviation (SD) and compared using a *t*-test. Qualitative variables were presented as frequency and percentage (%) and compared using the chi-square test. A two-tailed P value of less than 0.05 was considered statistically significant.

## 4. Results

**Figure 1.**
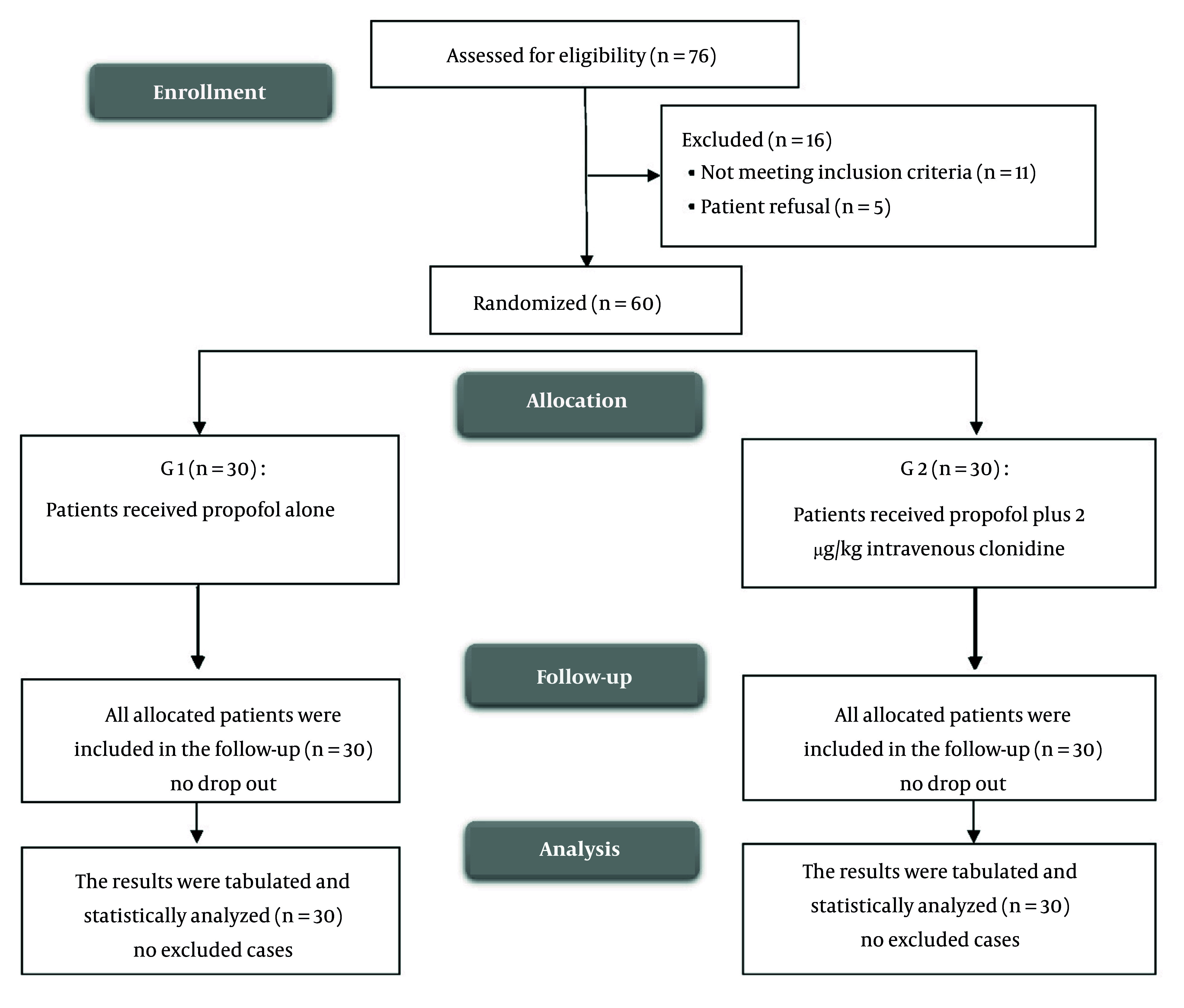
CONSORT flowchart of the enrolled patients

In this study, a total of 76 patients were assessed for eligibility. Of these, 16 were excluded: Eleven for not meeting inclusion criteria and 5 because they refused to participate. The remaining 60 patients were then randomized into two groups: Group 1 (n = 30) received propofol alone, and G2 (n = 30) received propofol plus 2 µg/kg intravenous clonidine. All 60 patients were included in the follow-up, with no dropouts from either group. The results were tabulated and statistically analyzed for both groups (Figure 1). 

Age, sex, BMI, ASA classification, indication for colonoscopy, and procedure time were not significantly different between the groups. The onset time of sedation was significantly earlier in G2 than in G1 (P = 0.001). The total amount of propofol consumed was lower in G2 than in G1 (P = 0.001) (Table 1). 

**Table 1.** Demographic Data, Indications of Colonoscopy, Onset Time of Sedation, Procedure Time, and Total Amount of Propofol Consumed During the Procedure in Both Groups ^a^

Variables	G1	G2	P-Value
**Age**	33.20 ± 10.08	33.73 ± 10.06	0.838
**Gender**			0.793
Male	17 (56.7)	18 (60)	
Female	13 (43.3)	12 (40)	
**BMI**	26.61 ± 3.40	26.17 ± 3.86	0.646
**ASA class**			0.118
I	10 (33.3)	16 (53.3)	
II	20 (66.7)	14 (46.7)	
**Indication for colonoscopy**			
Colorectal cancer screening	3 (10)	1 (3.3)	0.301
Gastrointestinal blood loss	14 (46.7)	17 (56.7)	0.438
Inflammatory bowel disease	13 (43.3)	12 (40)	0.793

Abbreviations: BMI: Body Mass Index; ASA, American Society of Anesthesiologists, G1, group 1; G2, group 2 .

^a^ Data are expressed as mean ± SD or No. (%).

**Figure 2.**
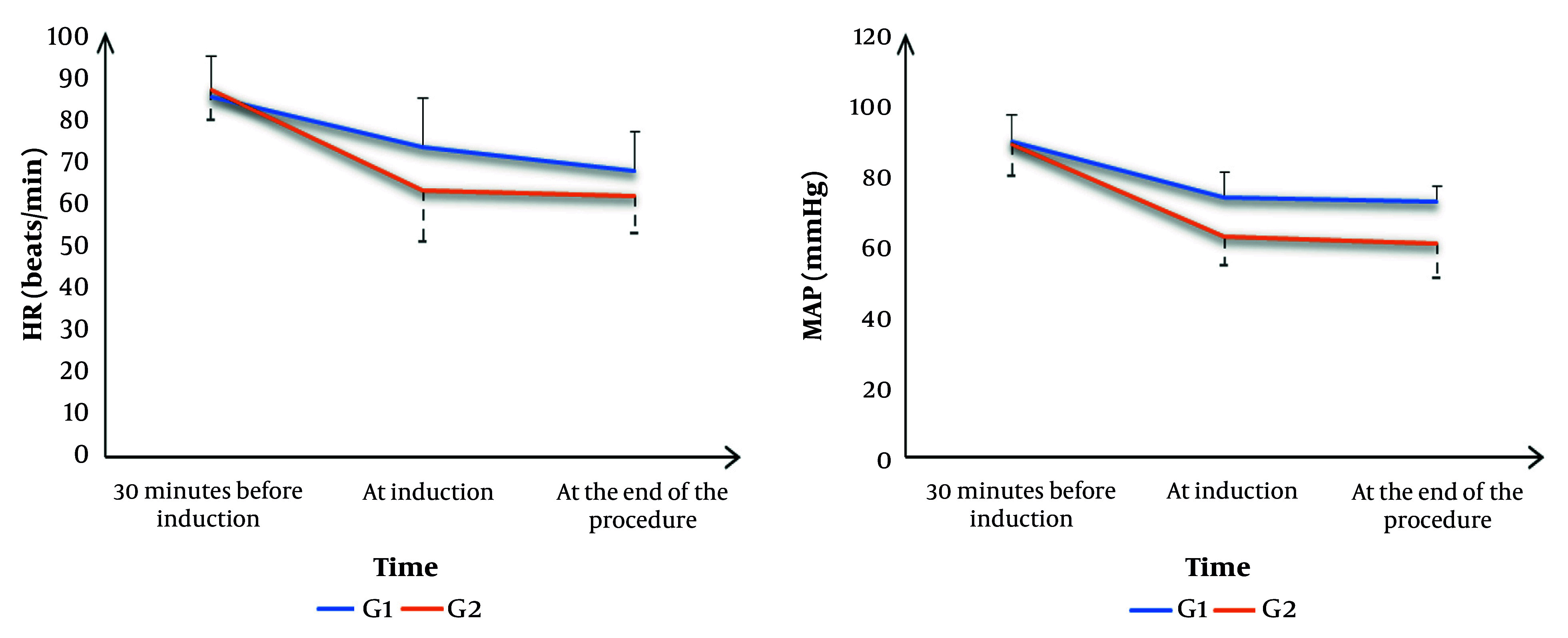
Hemodynamic records heart rate (HR) and mean arterial pressure (MAP) in both groups

Heart rate was not significantly different 30 minutes before induction between the groups. Heart rate was significantly lower at induction and at the end of the procedure in G2 compared to G1 (P = 0.002 and P = 0.015, respectively). Mean arterial pressure was not significantly different 30 minutes before induction between the groups. Mean arterial pressure was significantly lower at induction and at the end of the procedure in G2 compared to G1 (P = 0.001) (Figure 2). 

Patient satisfaction scores were significantly higher in G2 than in G1 (P = 0.042) (Table 2). 

**Table 2.** Patient Satisfaction Score in Both Groups ^a^

Patient Satisfaction Score	G1	G2	P-Value
**5**	12 (40)	21 (70)	0.042 ^b^
**4**	10 (33.3)	8 (26.7)	
**3**	7 (23.3)	1 (3.3)	
**2**	1 (3.3)	0 (0)	
**1**	0 (0)	0 (0)	

Abbreviation: G1, group 1; G2, group 2.

^a^ Values are expressed as No. (%).

^b^ Statistically significant P ≤ 0.05.

Bradycardia, Aldrete score at the end of the procedure, discomfort movement during the procedure, and PONV were not significantly different between the groups. The maximum change in MAP from baseline was significantly higher in G2 than in G1 (P = 0.001). The OAA/S after propofol induction and the Aldrete score in the post-anesthesia care unit (PACU) were significantly lower in G2 than in G1 (P = 0.015 and P = 0.001, respectively) (Table 3). 

**Table 3.** Recovery Scores and Complications in Both Groups ^a, b^

Variables	G1	G2	P-Value
**Bradycardia **	1 (3.3)	3 (10)	0.301
**Maximum change in MAP from baseline (%)**	18.93 ± 2.32	25.67 ± 2.82	0.001 ^c^
**OAA/S after a propofol induction dose**	3.50 ± 0.51	3.10 ± 0.71	0.015 ^c^
**Aldrete score at the end of the procedure**	5.07 ± 0.74	4.97 ± 0.76	0.609
**Aldrete score at PACU 15 minutes post-procedure**	9.43 ± 0.50	7.83 ± 0.75	0.001 ^c^
**Discomfort movement during the procedure**	3 (10)	1 (3.3)	0.301
**PONV**	3 (10)	1 (3.3)	0.301
**Onset time of sedation**	3.44 ± 1.16	2.41 ± 1.13	0.001 ^c^
**Procedure time**	23.10 ± 7.35	23.70 ± 7.91	0.762
**The total amount of propofol consumed**	9.61 ± 1.77	7.50 ± 1.33	0.001 ^c^

Abbreviations: MAP, mean arterial pressure; PACU, post anesthesia care unit; PONV, postoperative nausea and vomiting; OAA/S, observer's assessment of alertness/sedation ; G1, group 1; G2, group 2 .

^a^ Values are expressed as No. (%) or mean ± SD.

^b^ Discomfort movements during procedure refers to any physiological behavior requiring additional sedation or physical restraint, including purposeful movement, grimacing, or withdrawal responses.

^c^ Statistically significant P ≤ 0.05.

## 5. Discussion

This study aimed to evaluate the effectiveness, safety, and satisfaction outcomes of propofol alone versus propofol combined with clonidine for sedation during colonoscopy procedures. Our findings demonstrated several significant advantages of adding clonidine to propofol sedation, including faster sedation onset (2.41 ± 1.13 vs 3.44 ± 1.16 minutes, P = 0.001), reduced propofol requirements (22% reduction), improved patient satisfaction (70% vs 40% reporting highest satisfaction), and deeper sedation levels as measured by OAA/S scores. However, we also observed that the addition of clonidine was associated with lower Aldrete scores in the PACU, suggesting potentially prolonged recovery times.

These findings address important gaps in the current literature regarding optimal sedation protocols for colonoscopy. While propofol is widely used for colonoscopy sedation, the ideal combination of agents to maximize efficacy while minimizing side effects remains unclear. Our hypothesis that clonidine would enhance sedation quality while reducing propofol requirements was supported by the results, though the trade-off of longer recovery times warrants careful consideration.

The use of adjuvant medications in combination with propofol for sedation during colonoscopy procedures has been a subject of increasing interest in recent years, with studies exploring various agents to optimize sedation quality, reduce propofol consumption, and improve patient outcomes (8, 10, 11). One of the most significant findings in our research was a significant difference in the onset time of sedation among groups. Group 2, which received clonidine in addition to propofol, demonstrated a significantly shorter onset time of sedation (2.41 ± 1.13 minutes) compared to G1 (3.44 ± 1.16 minutes) (P = 0.001). This faster onset of sedation in the clonidine group is a clinically relevant finding that could improve efficiency in endoscopy units.

The more rapid onset of sedation observed with the addition of clonidine can be attributed to its pharmacological properties. Clonidine, an α_2_-adrenergic agonist, is known to have sedative effects and can potentiate the action of other sedative drugs (8). This synergistic effect with propofol likely contributes to the quicker onset of sedation. Interestingly, despite the difference in onset time, the overall procedure time was not significantly different among groups. This suggests that while clonidine may facilitate faster induction of sedation, it does not necessarily impact the duration of the colonoscopy procedure itself.

Another significant finding of our study was the marked reduction in total propofol consumption in the clonidine group. Patients in G2 required significantly less propofol (7.50 ± 1.33 mg/kg) compared to those in G1 (9.61 ± 1.77 mg/kg) (P = 0.001), representing a reduction of approximately 22% in propofol usage when clonidine was added to the sedation regimen. This reduction in propofol requirements aligns with the established propofol-sparing effect of adjuvant medications. Similarly, Moghadam et al. (12) found that patients premedicated with clonidine required significantly lower total doses of propofol compared to those who did not receive clonidine in a study involving patients undergoing elective below-knee surgeries.

The clinical implications of reduced propofol consumption are significant. Although propofol is an excellent sedative agent (11), it can be associated with dose-dependent adverse effects such as hypotension and respiratory depression. By reducing the total dose of propofol, adding clonidine may improve the safety of sedation for colonoscopy. This potential safety improvement is supported by a meta-analysis conducted by Zhang et al. (10), which reported that combining propofol with other agents had no significant effect on hypertension rates.

Our study revealed significant differences in hemodynamic parameters between the two groups. At induction, HR was significantly lower in G2 than in G1. This trend persisted until the end of the procedure, with G2 maintaining a lower HR than G1. Similarly, MAP was significantly lower in G2 compared to G1. This difference persisted until the end of the procedure, with G2 maintaining a lower MAP than G1. The observed reduction in HR and MAP in the clonidine group aligns with the known pharmacological effects of clonidine, which, as an α-adrenergic agonist, decreases the release of sympathetic signals from the central nervous system, leading to decreased sympathetic transmission to the heart and blood vessels and increased vagal tone. Clinically, it lowers MAP, HR, and peripheral resistance (13, 14).

Beyond bradycardia and hypotension, no additional side effects such as dry mouth, dizziness, or excessive sedation were reported by participants or observed during follow-up. Our study revealed significant differences in patient satisfaction scores among groups (P = 0.042). In G2, which received clonidine in addition to propofol, 70% of patients reported the highest satisfaction score of 5, compared to only 40% in G1. Furthermore, only 3.3% of patients in G2 reported a satisfaction score of 3 or lower versus 26.6% in G1. These results indicate that incorporating clonidine into propofol sedation may enhance patient satisfaction during colonoscopy procedures. The improved satisfaction scores in the clonidine group could be attributed to several factors: anxiolytic properties, deeper sedation, analgesic effects, and a smoother sedation profile (15, 16).

The similar rates of PONV between the groups (10% in G1 vs. 3.3% in G2) are noteworthy. Although the disparity was not statistically significant, there was a trend toward lower PONV in the clonidine group. This aligns with studies showing that propofol, compared to traditional sedatives, significantly reduces nausea and vomiting (11). Additionally, premedication with clonidine may reduce PONV (17, 18).

As mentioned earlier, the maximum change in MAP from baseline was significantly higher in G2 (25.67 ± 2.82%) versus G1 (18.93 ± 2.32%) (P = 0.001). This greater fluctuation in MAP in the clonidine group is likely due to the combined vasodilatory effects of clonidine and propofol (13, 14).

The depth of sedation and recovery characteristics are crucial to any sedation protocol. To evaluate these parameters, our study utilized the OAA/S scale and the Aldrete score. Our study demonstrated that the OAA/S score after propofol induction was significantly lower in G2 (3.10 ± 0.71) than in G1 (3.50 ± 0.51) (P = 0.015). This finding indicates that patients in the clonidine group achieved a deeper level of sedation with the same induction dose of propofol. This is consistent with the known sedative properties of clonidine and its ability to potentiate the effects of other sedative agents (19, 20).

Interestingly, while the Aldrete score at the end of the procedure was not significantly different between the groups, there was a significant difference in the Aldrete score in the PACU. Group 2 had a significantly lower Aldrete score in the PACU (7.83 ± 0.75) compared to G1 (9.43 ± 0.50) (P = 0.001). The comparable Aldrete scores at the end of the procedure indicate that both sedation regimens resulted in similar immediate recovery. However, the lower Aldrete scores in the PACU for the clonidine group suggest a potentially longer recovery period, whereas propofol alone allows for a quicker recovery (20).

The study used a fixed clonidine dose of 2 μg/kg to maintain consistency and facilitate direct comparisons. A dose-response analysis was beyond the scope of this study but will be explored in future research to optimize dosing strategies for different patient demographics.

The study's limitations include a small sample size, reducing generalizability; a single-center design, restricting clinical applicability; and limited follow-up, preventing long-term outcome assessment. The fixed clonidine dose of 2 μg/kg may not suit all patients, as individual pharmacokinetic variations due to age, metabolism, and medical conditions were unaccounted for in this study. Additionally, the lack of Bispectral Index (BSI) monitoring may have limited the ability to precisely assess the depth of sedation and the potential for intraoperative awareness. Future research should address these constraints by expanding cohort diversity, conducting multi-center studies, extending follow-up periods, and investigating personalized dosing strategies.

### 5.1. Conclusions

Incorporating clonidine into propofol sedation for colonoscopy results in several significant changes compared to propofol sedation alone. These include a faster onset of sedation, reduced propofol consumption, lower HR and MAP during the procedure, improved patient satisfaction, and deeper levels of sedation but with prolonged recovery times.

## Data Availability

Data is available upon reasonable request from corresponding author.

## Clinical Trial Registration Code:

NCT06507410.
